# Development of Randomly Amplified Polymorphic DNA Based SCAR Marker for Identification of *Ipomoea mauritiana* Jacq (Convolvulaceae)

**DOI:** 10.1093/ecam/neq023

**Published:** 2011-02-20

**Authors:** Kambiranda Devaiah, Subramani Paranthaman Balasubramani, Padma Venkatasubramanian

**Affiliations:** ^1^Centre for Pharmacognosy, Pharmaceutics and Pharmacology, Foundation for Revitalization of Local Health Traditions (FRLHT), via Yelhanka, Bangalore 560106, India; ^2^Center for Viticulture and Small Fruit Research, Florida A & M University, 6505 Mahan Drive, Tallahassee, FL 32317, USA

## Abstract

Vidari is an Ayurvedic herbal drug used as aphrodisiac, galactagogue and is also used in the preparation of *Chyavanaprash*. Tubers of *Ipomoea mauritiana* Jacq. (Convolvulaceae), *Pueraria tuberosa* (Roxb. ex Willd.) DC (Fabaceae), *Adenia hondala* (Gaertn.) de Wilde (Passifloraceae) and pith of *Cycas circinalis* L. (Cycadaceae) are all traded in the name of Vidari, creating issues of botanical authenticity of the Ayurvedic raw drug. DNA-based markers have been developed to distinguish *I. mauritiana* from the other Vidari candidates. A putative 600-bp polymorphic sequence, specific to *I. mauritiana* was identified using randomly amplified polymorphic DNA (RAPD) technique. Furthermore, sequence characterized amplified region (SCAR) primers (IM1F and IM1R) were designed from the unique RAPD amplicon. The SCAR primers produced a specific 323-bp amplicon in authentic *I. mauritiana* and not in the allied species.

## 1. Introduction

Vidari is one of the popular plant drugs of Ayurveda and is a component of many popular and highly traded Ayurvedic formulations like *Chyavanaprash*, an ancient Indian dietary supplement. The Ayurvedic Pharmacopoeia of India correlates “Vidari” to tubers of *Pueraria tuberosa* (Roxb. ex Willd.) DC (Fabaceae) and *Ipomoea mauritiana* Jacq. (Convolvulaceae) as “Kshiravidari” [1]. However, a recent report by Venkatasubramanian et al. [2] indicates that as per Ayurvedic descriptions they both have similar properties and can be substituted by each other. Several herbal medicine manufacturing units also use the giant potato or *I. mauritiana* (Syn. *I. paniculata* or *I. digitata)* as Vidari instead of *P. tuberosa*. Vidari is useful as aphrodisiac, cardiotonic, demulcent, diuretic, refrigerant and galactogogue [3]. It is also used in emaciation, enteric fever and spermatorrhea [4]. The annual trade volume of Vidari is *∼*500–1000 Metric Tonnes [5].

*Ipomoea mauritiana* is a branched perennial climber with large tuberous tap roots and glabrous stems and branches; leaves palmately 5–7 lobed; flowers purple, in pedunculate corymbose axillary panicles; fruits ovoid, four-celled and four-valved capsules, surrounded by enlarged fleshy sepals, seed clothed with many long tawny cottony hairs. The root tubers exude milky, sticky, latex and exhibits annual rings when cut. This species is widely naturalized in tropical parts of the world [6]. Taraxerol, taraxerol acetate, umbelliferone, *β*-sitosterol, scopoletin and 7-*O*-*β*-d-glycopyranosyl scopoletin (Scopolin) have been isolated from the methanol extract of the tubers [7]. The roots are used to increase appetite, as a galactagogue, in rejuvenative medicine, as a stimulant, carminative and tonic [8]. Alcohol extract of tubers is stimulant as well as depressant, and has convalescent effect on central nervous system [9, 10].

Apart from tubers of *P. tuberosa* and *I. mauritiana*, tubers of *Adenia hondala* (Gaertn.) de Wilde (Passifloraceae) and the pith of *Cycas circinalis* L. (Cycadaceae) are also traded in the name of Vidari [5]. The Ayurvedic Pharmacopoeia of India stipulates macro–microscopic evaluation and chemical profiling of the botanical materials for quality control and standardization [1]. Use of macro, micro and chemical techniques for authentication has its advantages and disadvantages [11], while molecular markers, once standardized is fool-proof, easy and objective [12].

Molecular genetic tools like barcoding, random amplified polymorphic DNA (RAPD) and sequence characterized amplified region (SCAR) markers are reliable methods for quality control of herbal materials. Fingerprints obtained by RAPD can be employed for identification of raw drug at the molecular level [13], however since it is difficult to reproduce these fingerprints they are preferentially converted to SCAR markers. Molecular marker technology proves to be valuable tool not only for genotyping of medicinal plants but also for detecting adulterations and substitutions in herbal medicines [14–17].

In our study, we have developed RAPD profile of *I. mauritiana, P. tuberosa, A. hondala* and *C. circinalis*. Subsequently, a unique DNA sequence identified from the RAPD profile of *I. mauritiana* was used to develop a species-specific SCAR marker. This marker is useful to distinguish *I. mauritiana* from its allied species traded as Vidari.

## 2. Methods

### 2.1. Plant Material

Field and market samples were collected from different geographical regions across India. Plant samples were authenticated by qualified field botanist and an Ayurvedic practitioner at Foundation for Revitalization of Local Health Traditions (FRLHT). The voucher samples were deposited in the Herbarium and Raw Drug Repository (FRLHT, Bangalore, India). The details of the plant samples used in this study are presented in Table 1.

### 2.2. Reagents and Chemicals

CTAB (20% (w/v)), 1 M Tris-HCl (pH 8), 5 M EDTA (pH 8), 5 M NaCl, 3 M sodium acetate, ethanol, chloroform-isoamyl alcohol (24 : 1 (v/v)), polyvinylpyrrolidone (PVP) (40 000 mol*·*wt) (Sigma), and *β*-mercaptoethanol. All the chemicals used in the experiments were of analytical grade. Enzymes (Taq Polymerase and RNase A), Taq buffer, MgCl_2_ and dNTPs for polymerase chain reaction (PCR) amplification were purchased from Bangalore Genei (Bangalore, India).

### 2.3. DNA Extraction

Dried raw plant materials were chopped into small pieces and powdered using liquid nitrogen in mortar and pestle. Samples were later homogenized using CTAB extraction buffer and processed as described in the protocol of Milligan [18]. The extracted DNA was treated with 5 *μ*L of RNase A (10 mg/mL) to remove any contaminating RNA. Purity and yield of DNA were checked using UV Spectrophotometer (UV Pharmaspec 1700, Shimadzu, Tokyo, Japan) by calculating the A_260_/A_280_ [19].

### 2.4. RAPD Reaction

The PCR was performed by adding 25 ng of plant DNA, 2 mM dNTP mix, 30 pM of primer, 2.5 *μ*L of 10× PCR buffer, 2.5 mM MgCl_2_, 2.0 U of Taq DNA Polymerase (Bangalore Genei, India) and made up to 25 *μ*L with distilled water. The reaction was performed in a thermal cycler (Eppendorf Master Cycler Gradient, Hamburg, Germany). Reaction conditions were as follows: 94°C—3 min, 40 cycles of 94°C—30 s, 36°C—30 s, 72°C—1 min and a final extension of 72°C for 10 min. PCR products were resolved in a horizontal gel electrophoresis system (Classic CSSU1214, Thermo Electron, MA, USA) along with standard 100-bp ladder and *λ* DNA EcoR I/HindIII double digest (Bangalore Genei, India) on a 1.2% agarose, 0.5× TBE gel. The DNA was stained with ethidium bromide (0.5 *μ*g/mL). The gel was visualized under UV radiation in a Camag Reprostar (022.9610, CAMAG, Muttenz, Switzerland) and digitally photographed (Canon, Tokyo, Japan). RAPD was performed with 40 random decamer primers (OPA 1–20 and OPF 1–20) obtained from Operon Technologies (Alameda, CA, USA). Electrophoretic profiles were analyzed for polymorphism based on the presence and absence of DNA bands on agarose gel. Confirmation of results was done using at least three different samples of each species. Experiment was repeated at least three times to confirm species-specific fingerprinting pattern and reproducibility.

### 2.5. Cloning and Sequencing of the Polymorphic Band

The putative marker amplified by the random primer OPA 18 was excised from 1.2% agarose gel with sterile gel slicer and purified using the QIAquick Gel extraction kit (Qiagen, Maryland, USA). The purified fragment was reamplified using the primer OPA 18 with the same PCR conditions, except for the final extension, which was increased to 20 min to facilitate cloning into TA cloning vector. About 2 *μ*L of the purified DNA was ligated into a pDRIVE vector and transformed into Qiagen EZ competent cells following the manufacturer's instructions (Qiagen, Maryland, USA). Ten distinct white colonies were picked from the LB/ampicillin/X-gal/IPTG plate and recombinant plasmid was isolated from each overnight grown colony. The presence of insert was confirmed by reamplifying the DNA using the OPA 18 under similar RAPD conditions. The recombinant plasmid was purified using the Quicklyse Mini prep kit (Qiagen, Maryland, USA). DNA insert was sequenced on an ABI 310 Automated Sequencer (Applied Biosystems, CA, USA) using vector specific universal promoter primers SP6 (5′-CATACGATTTAGGTGACACTATAG-3′) and T7 (5′-TAATACGACTCACTATAGGG-3′) by Bioserve Biotechnologies (Hyderabad, India).

### 2.6. SCAR Primer Designing and Validation

Based on the sequence of unique RAPD amplicon a pair of SCAR oligonucleotide primers (IM 1F and IM1R), which could amplify *∼*323 bp of the genomic DNA of *I. mauritiana* was designed (Table 2). The oligos were custom-synthesized by Bioserve Biotechnologies (Hyderabad, India). The SCAR primer pair was used for PCR amplifications of genomic DNA from all the Vidari species. The reaction mixture contained 25 ng of plant DNA, 0.6 mM dNTP mix, 30 pM of primer, 2.5 *μ*L of 10× PCR buffer, 1.5 mM MgCl_2_, 1.0 U of Taq DNA Polymerase (Bangalore Genei, India) and made up to 25 *μ*L with distilled water. PCR conditions for amplification using SCAR primers were optimized as: 95°C for 3 min; 35 cycles of 95°C for 35 s, 60°C for 30 s, 72°C for 45 s and a final extension at 72°C for 3 min. The SCAR primers were validated using three accessions each of *I. mauritiana*, *P. tuberosa*, *A. hondala* and *C. circinalis* while repeating each experiment three times.

## 3. Results

### 3.1. DNA Extraction and RAPD

**Figure 1:**
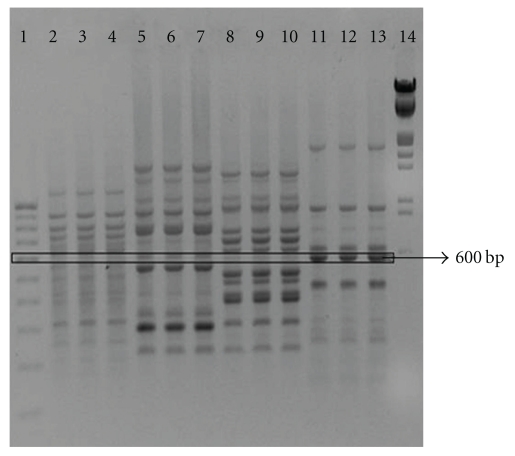
RAPD pattern of Vidari candidates. Lane: 1—Standard DNA marker (100-bp DNA ladder); 2–4—*A. hondala*; 5–7—*C. circinalis*; 8–10—*P. tuberose*; 11–13—*I. mauritiana*; 14—Standard DNA marker (*λ* DNA EcoR I/HindIII double digest).

High molecular weight genomic DNA was isolated from the fresh as well as dried plant samples. The DNA extraction procedure yielded 400–600 ng of DNA per 100 mg of tissue. An absorbance (A_260_/A_280_) ratio of 1.6–1.8 indicated insignificant levels of contaminating proteins and polysaccharides. All the 40 random decamer primers used for screening, produced distinct, reproducible fingerprint of *I. mauritiana, P. tuberosa, C. circinalis* and *A. hondala*. OPA 18 consistently amplified an intense 600-bp band that was unique to *I. mauritiana* samples and invariant with all the three accessions of this species tested (Figure 1). This 600-bp amplicon was not observed in the other three species under study.

### 3.2. Cloning and Sequencing of Polymorphic Band

**Figure 2:**
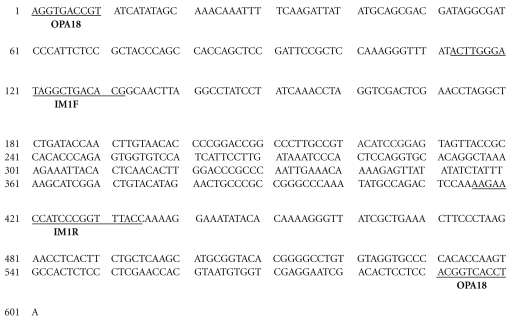
Sequence of 600-bp polymorphic region of *I. mauritiana*, showing binding sites of RAPD (OPA 18) marker and SCAR Marker (IM1F and IM1R).

*Ipomoea mauritiana* specific 600-bp polymorphic band was cloned into pDRIVE cloning vector and transformed into Qiagen EZ competent cells. The selected white colonies contained the required recombinant construct as was confirmed by reamplification with OPA18. The recombinant construct was sequenced using universal sequencing primers SP6 and T7. The sequencing reaction yielded a 601-bp stretch with the purified band from *I. mauritiana* (A_371; C_211; G_212; T_323) (Figure 2).

### 3.3. Analysis of Sequence and SCAR Primer Designing

The DNA sequence of the polymorphic band of *I. mauritiana* was submitted to Genebank (Accession number: EF624353; http://www.ncbi.nlm.nih.gov/). Homology searches were performed within Genebank's non-redundant database using the Basic Local Alignment Search Tool (BLAST) algorithm at http://www.ncbi.nlm.nih.gov/BLAST/ of the National Center for Biotechnology Information (NCBI), with the program BLASTN. BLAST results revealed that the sequence did not have similarity with any known nucleotide sequences. Based on the sequence of the unique polymorphic band, SCAR primers were designed using NCBI primer blast tool (http://www.ncbi.nlm.nih.gov/tools/primer-blast/). Primer designing was done with high stringency, so as to give specific amplification only with *I. mauritiana* samples (Table 2).

### 3.4. Validation of SCAR Primers

**Figure 3:**
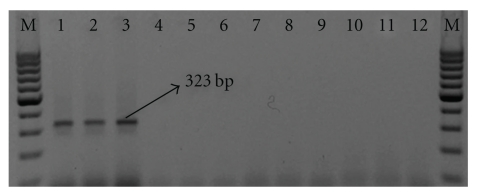
Validation of *I. mauritiana* specific SCAR marker. Lane: M—Standard DNA marker (100-bp DNA ladder); 1–3—*I. mauritiana*; 4–6—*A. hondala*; 7–9—*C. circinalis*; 10–12—*P. tuberose*.

The designed SCAR primer pair (IM1F and IM1R; Table 2) was used to amplify the genomic DNA from *I. mauritiana*, *C. circinalis*, *A. hondala* and *P. tuberosa*. A single, distinct and brightly resolved band of 323 bp was obtained only with the accessions of *I. mauritiana*, while no amplification product was obtained with *P. tuberosa, C. circinalis* and *A. hondala* (Figure 3). Reduction of the annealing temperatures did not generate any fragment other than the SCAR, confirming the specificity of the SCAR primer for *I. mauritiana*.

## 4. Discussion

Herbal medicine has been enjoying renaissance among the customers throughout the world. Use of indigenous drugs from plant origin forms a major part of complementary and alternative medicine/traditional medicine (CAM/TM). One of the impediments in the acceptance of herbal formulations is the lack of standardization and quality control profiles. Due to the complex nature and inherent variability of the chemical constituents of plant-based drugs, it is difficult to establish quality control standards. Adulteration of market samples remains a major problem in domestic and export markets due to confusing nomenclature and lack of botanical identification of traded raw drugs [20, 21]. Active chemical composition of the herbal formulation depends on several factors including the use of exact species [22]. According to World Health Organization general guidelines for methodologies on research and evaluation of TMs, first step in assuring quality, safety and efficacy of TMs is correct identification [23].

*Panax* (ginseng) is a representative genus of medicinal herbs which was subjected to several methods of DNA analysis. The methods, applied by several research groups, included PCR-RFLP, AFLP, RAPD, SSR, sequencing of rDNA-ITS region and DNA barcoding. This is an example of how accurate methods for authenticating medicinal plants are necessary to modernize and standardize herbal medicine [24, 25].

RAPD is rapid, simple and can be performed even in the absence of prior genetic information of the plant. The fingerprint patterns obtained are consistent irrespective of physical form, age or agroclimatic source [26].

Many technical disadvantages associated with RAPD have, however, raised questions on its fidelity as a genetic marker technique and prevented its widespread use in recent years. The reproducibility of RAPD is affected by DNA quality, primer and template concentration, different thermocyclers and even different sources of DNA polymerase [15]. Therefore, subsequent conversion of RAPD to SCAR markers, by developing longer, hence more specific primers from RAPD sequences, has significantly improved the reproducibility and reliability of PCR assays [27, 28]. Species-specific SCAR markers have been used in several studies to differentiate important Indian medicinal plants from their close relatives or adulterants [17, 18]. SCAR marker for discriminating *Anthricus sylvestris*, an adulterant of *Peucedanum praeruptorum* and *Peucedanum decursivum* (syn. *Angelica decursiva)* used widely in Chinese, Japanese and Korean TM has been reported [28]. Furthermore, development of species-specific DNA markers can increase industrial application of the molecular techniques [27].

**Figure 4:**
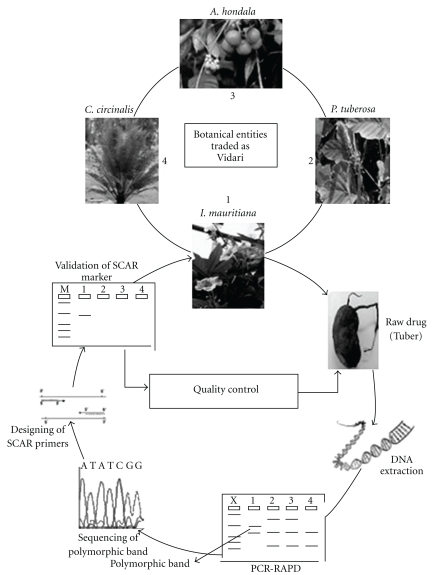
Diagrammatic representation of RAPD-SCAR marker development to authenticate *I. mauritiana* Jacq.

In our RAPD analysis, significant genetic polymorphism was observed among *I. mauritiana* and its adulterants and substitutes. The SCAR primers designed using this sequence variation was found to be specific for *I. mauritiana*, making the technique more stringent and specific when compared with RAPD marker. The SCAR primers can be used to authenticate the identity of *I. mauritiana* from other traded “Vidari” candidates like *C. circinalis*, *A. hondala* and *P. tuberosa*. The complete process involved in developing RAPD-based SCAR marker for authentication of *I. mauritiana* is summarized in Figure 4. Such efficient, precise and sensitive techniques are required in quality control of raw drugs in the herbal industry.

##  Funding

“Centre of Excellence” grant from Ministry of Environment and Forests, Government of India.

## Figures and Tables

**Table 1:** Details of the plant samples used in the study.

Name of the plant species	Accession number	Place of collection
*Pueraria tuberosa*	L/07/10/028	Belgaum, Karnataka
	L/07/02/032	Pune, Maharastra^a^
	L/04/07/009	Not Available

*Ipomoea mauritiana*	L/08/08/014	Wayanad, Kerala
	L/08/08/018	Kollukayal, Kerala
	L/08/08/019	Kollukayal, Kerala

*Adenia hondala*	L/07/08/009	Thiruinelli, Kerala
	L/07/08/025	Kozhikode, Kerala
	L/09/02/006	Kambamda, Kerala

*Cycas circinalis*	L/02/01/006	Not Available
	L/07/09/006	Bangalore, Karnataka^a^
	L/07/09/007	Bangalore, Karnataka^a^

^a^Market sample authenticated by botanist.

**Table 2:** Details of the *I. mauritiana* specific SCAR marker designed from the 600-bp polymorphic sequence.

Name of random decamer primer used	Sequence of random decamer primer (5′-3′)	Name of the SCAR primer	Sequence of the SCAR primer (5′-3′)	Annealing temperature (°C)
OPA18	AGGTGACCGT	IM1F	ACTTGGGATAGGCTGACACG	60
		IM1R	GGTAAACCGGGATGGTTCTT	60
